# A VigiBase descriptive study of fluoroquinolone induced disabling and potentially permanent musculoskeletal and connective tissue disorders

**DOI:** 10.1038/s41598-021-93763-y

**Published:** 2021-07-13

**Authors:** Madalina Huruba, Andreea Farcas, Daniel Corneliu Leucuta, Camelia Bucsa, Mariana Sipos, Cristina Mogosan

**Affiliations:** 1 Department of Pharmacology, Physiology and Physiopathology, Faculty of Pharmacy, Iuliu Haţieganu” University of Medicine and Pharmacy, Cluj-Napoca, Romania; 2 Drug Information Research Center, “Iuliu Hatieganu” University of Medicine and Pharmacy, Pasteur Street no 6A, Cluj-Napoca, Romania; 3 Department of Medical Informatics and Biostatistics, Iuliu Hatieganu” University of Medicine and Pharmacy, Cluj-Napoca, Romania

**Keywords:** Risk factors, Signs and symptoms

## Abstract

Recent drug safety concerns described fluoroquinolone (FQ)-induced serious musculoskeletal reactions. The objective of this study was to characterize reports with FQ-associated disabling musculoskeletal disorders, from VigiBase. The analysis included all FQ-induced musculoskeletal and connective tissue disorders adverse drug reaction (ADR) reports (up to July-2019), (disabling/incapacitating, or recovered/resolved with sequelae or fatal). We described aspects like reporter, suspected FQs, ADRs, associated corticosteroid therapy. We also looked into the disproportionality data in terms of proportional reporting ratio (PRR) and information component (IC) values. A total of 5355 reports with 13,563 ADRs and 5558 FQs were reported. The majority of reports were for patients aged 18–64 (62.67%), and the female gender prevailed (61.76%). Consumers reported almost half (45.99%), with a peak in reporting rates in 2017. Top reported ADRs were arthralgia (16.34%), tendonitis (11.04%), pain in extremity (9.98%), tendon pain (7.63%), and myalgia (7.17%). Top suspected FQs were levofloxacin (50.04%), ciprofloxacin (38.41%), moxifloxacin (5.16%), ofloxacin (3.17%) and norfloxacin (1.01%). For these, FQs-ADR association was supported by the disproportionality analysis. Corticosteroids were associated with about 7% of tendon related reports. The results augment the existing data on FQs safety concerns, specifically their potential effect on the musculoskeletal system.

## Introduction

The burden of adverse drug reactions (ADRs) on public health is high^1^. It represents an important medical issue resulting in 3–7% of all hospital admissions, being associated with a marked increase in morbidity and mortality^2^. Muscle disorders may be less promptly recognized as compared to the skin or allergic reactions; however, the musculoskeletal system can be a target for ADRs. Musculoskeletal ADRs can be temporarily disabling (e.g., muscle cramps), but can also be serious and life-threatening (e.g., rhabdomyolysis). Increased awareness of drug-induced adverse effects on the musculoskeletal system has been recognized during the last few years. Recent drug safety issues pointed out to serious or uncommon musculoskeletal reactions, including tendon rupture associated with fluoroquinolones (FQs), especially in older patients, renal failure and concomitant corticosteroid therapy^1–3^.

Concern regarding the impact of FQs on the musculoskeletal safety exists from preclinical studies, indicating cartilage injury in weight-bearing joints, dependent on dose and duration of therapy^4,5^. A class of antibiotics with broad-spectrum antimicrobial activity^6^, FQs have been used in clinical practice for up to 40 years, frequently prescribed due to their good tolerability^7^. The newer generation agents have demonstrated effectiveness in the treatment of respiratory tract infections^8^. While the efficacy of FQs has improved from the first (e.g., nalidixic acid) to the third generation (e.g., moxifloxacin), careful evaluation of their benefit-risk profile still needs to be in focus. Fluoroquinolones are increasingly being recognized as a cause of disabling ADRs such as tendinitis and tendon rupture^9,10^. Ciprofloxacin and levofloxacin, although generally safe for therapeutic use, may induce tendinopathic complications such as tendinitis^11^. When compared to other antibiotics, the relative risk of FQ-induced tendinitis was 3.7, as reported by a post-authorization cohort study^12^, while a different study observed a relative risk of 8.0 for tendinitis and tendon rupture in lung transplant patients prescribed ciprofloxacin^10^.

A safety review of disabling and potentially permanent serious side effects of systemic FQs involving the peripheral and central nervous system as well as tendons, muscles, and joints, was finalized by the U.S. Food and Drug Administration (FDA) in 2016. This evaluation resulted in the restriction of use and FQs being reserved for patients with no other treatment alternative^13^. In 2017, the European Medicines Agency (EMA) initiated an evaluation of FQ-associated safety concerns, aiming to assess the impact of these concerns on the benefit-risk balance of quinolone and FQ-containing medicinal products. As a result, the marketing authorization of medicines containing cinoxacin, flumequine, nalidixic acid, and pipemidic acid was suspended, and the use of the remaining FQs was restricted in the European Union^3^.

The objective of this retrospective study was to characterize individual case safety reports (ICSRs) with an FQ as the suspected drug, resulting in disabling musculoskeletal and connective tissue disorders, from VigiBase, the unique World Health Organization (WHO) global database of ICSRs.

## Methods

### Data source

VigiBase, a database maintained by the Uppsala Monitoring Centre (UMC), with more than 19 million ICSRs submitted by national pharmacovigilance centers from 1967 up to May 2019, was used as the data source for this study. ICSRs include information on patient characteristics, ADRs, suspected drugs, and additional information relevant to the report, such as seriousness, reporter type, reporting year, and region. The reports in VigiBase originate from multiple sources: different countries and different types of reporters (e.g., physicians, pharmacists, other health care professionals (HCPs), patients), and are generally notified post-marketing. As a result of the source diversity, a variation exists regarding the amount of information in each report. Also, the probability that the suspected adverse effect is drug-related is not the same in all cases.

All musculoskeletal and connective tissue disorders ADR reports (i.e., Medical Dictionary for Regulatory Activities [MedDRA] Preferred terms [PTs] grouped under the System Organ Class [SOC] *Musculoskeletal and connective tissue disorders* [further referred to as "musculoskeletal"]) associated with an FQ (ATC code: J01M) until 2019–07-01, were extracted from the UMC global database and were received deduplicated. In VigiBase, reactions are allocated codes according to the latest versions of the hierarchical structures of MedDRA (i.e., version 22.0 at the time of the search)^14^.

### Data selection and analysis of case reports

Our analysis included all cases where an FQ was suspect (i.e., “WHO Drug preferred base name”), with seriousness criteria as *disabling/incapacitating*, or outcome *recovered/resolved with sequelae* or *fatal*. We excluded all reports that had additional drugs suspected to have caused an ADR of the evaluated SOC (i.e., other drugs with *basis* suspect in relation to a PT grouped under the upper mentioned SOC). In the absence of the respective narratives, we also excluded cases with seriousness criteria *congenital anomaly/birth defect* in addition to the seriousness criteria of interest. Lastly, we also excluded duplicates of the cases numbered twice due to having both the seriousness and outcome criteria of interest. For the selected reports we described aspects like reporter, region, year of the report, seriousness, suspected FQs, and route of administration, ADRs and dechallenge/re-challenge outcome.

With respect to *serious* criteria, (i.e., “yes” or “no”), a positively *serious* criterion is based on patient/event outcome or action posing a threat to a patient's life or functioning. This information becomes of critical importance since it can indicate if due to the nature of the reaction (“serious”) or due to the significant, unexpected information the report provides, expedited reporting is justified in terms of regulatory actions^15^. In VigiBase, whether or not the report was “serious” is a given unique criterion applicable to the overall report. *Seriousness* on the other hand, although linked with a specific ADR, is treated in bulk per report. Reports considered not “serious” had *seriousness* either unknown or “disabling/incapacitating”—but presumably not representing a threat to the patient’s life or functioning.

Further descriptive analysis was performed for the topmost reported FQs and ADRs for which we looked into time to onset and duration of the reaction. For the top reported FQs with oral or parenteral administration we selected the musculoskeletal disorders related to the tendon (i.e., PTs: tendon discomfort, tendon disorder, tendon pain, and tendonitis), for which we looked into potential risk factors such as age, gender, dose, concomitant/interacting corticosteroids (i.e., a search performed for all corticosteroids for systemic use, within ATC code: H02A, that were concomitant or interacting), treatment duration. We considered a low/high dose any amount below/above WHO's Defined Daily Doses (DDD) for each FQ^16^ and a long duration of therapy to be more than 7 days.

The present analysis refers to ADRs in terms of MedDRA PTs. More than one FQ and/or ADR could be reported per ICSR.

Descriptive statistics were used to summarize the baseline characteristics of the reports and reactions.

### Disproportionality data analysis

Disproportionality data analysis was performed overall, for all musculoskeletal FQ-related ADRs. Proportional Reporting Ratio (PRR) and information component (IC) (specifically developed and validated by UMC as a flexible, automated indicator value for disproportionate reporting) with their 95% confidence intervals (CIs) were used as provided by the UMC. A PRR025 (lower end of the 95% CI for PRR) value > 1 associated with ≥ 5 cases was considered a positive association between the FQ and the ADR^17,18^. An IC025 value (lower end of the CI for IC) ≥ 0 was considered a positive FQ-ADR association. Details on the IC and IC025 calculations were previously described^19^.

## Results

**Figure 1:**
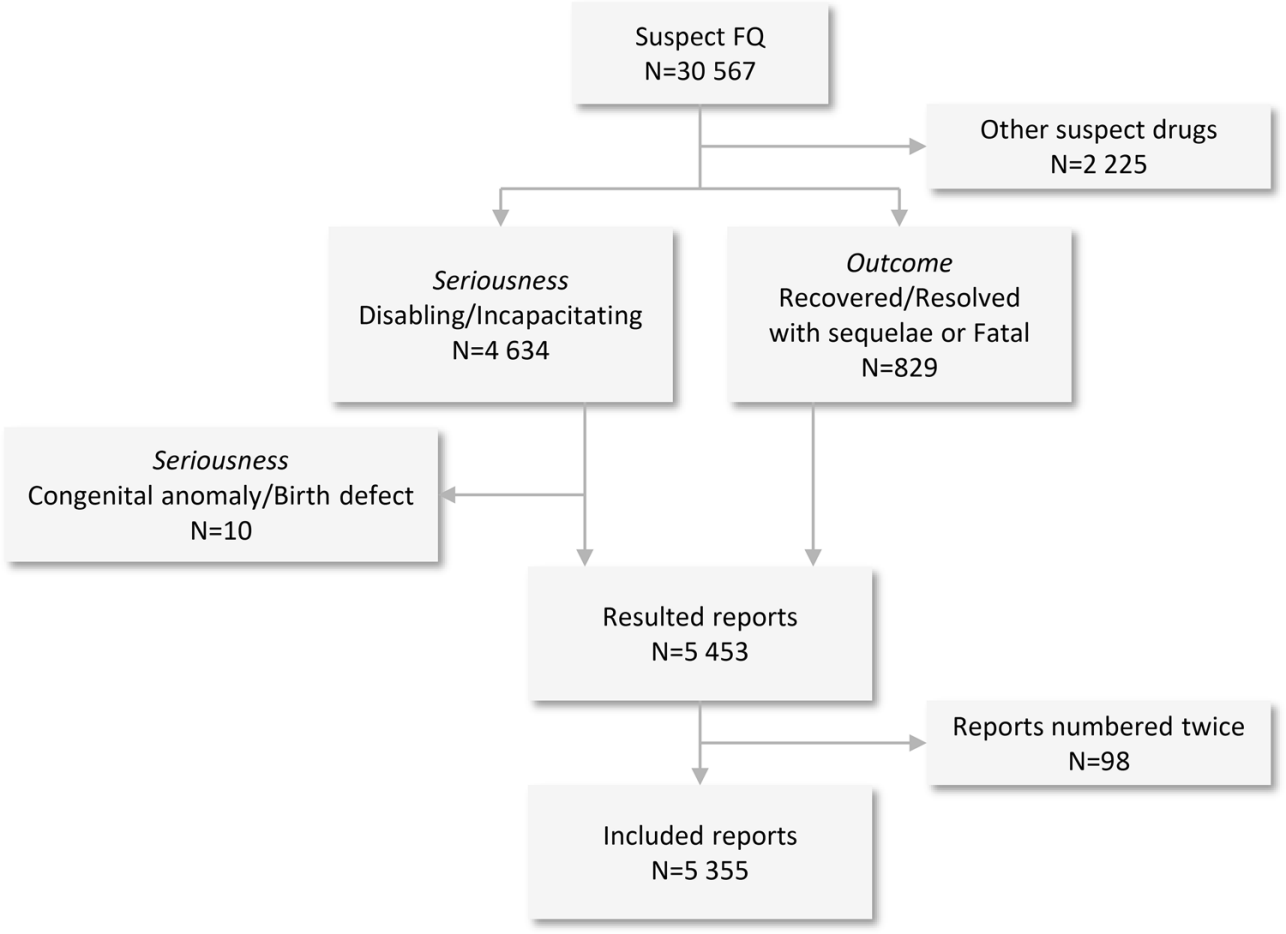
Overview of inclusion and exclusion criteria.

Out of a total number of 30,567 reports with an FQ suspect of causing musculoskeletal disorders, a total of 5355 were included in the analysis based on the inclusion/exclusion criteria. (Fig. 1).

**Figure 2:**
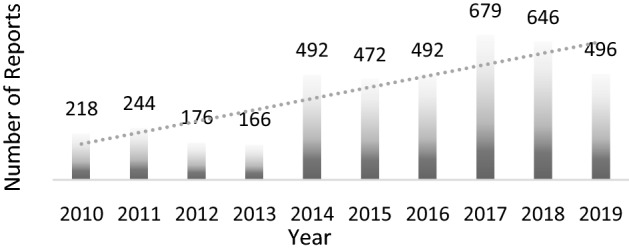
Year trends of reporting.

The majority of reports were for patients between 18 and 64 years of age (62.67%), and the female gender prevailed (61.76%). Most of them originated from America (74.21%) and were generally reported by consumers/non-HCPs (45.99%) (Table 1). A peak in reporting rates was observed in 2017. The year trends of reporting are presented in Fig. 2.

**Table 1:** Characteristics of musculoskeletal and connective tissue disorders reports for fluoroquinolones.

	Number of reports N = 5355, n (%)		n (%)
**Sex**		Reporter*	**n = 5512 (100.00)**
Male	1957 (36.54)	HCP	1428 (25.91)
Female	3307 (61.76)	Consumer	2535 (45.99)
Unknown	91 (1.70)	Other	35 (0.63)
		Unknown	1514 (27.47)
**Age, years**		**ADR**^**†**^	**n = 13,563 (100.00)**
< 18	51 (0.95)	Arthralgia	2216 (16.34)
18–64	3356 (62.67)	Tendonitis	1498 (11.04)
≥ 65	1251 (23.36)	Pain in extremity	1353 (9.98)
Unknown	697 (13.02)	Tendon pain	1035 (7.63)
**UN Continent**		Myalgia	973 (7.17)
America	3974 (74.21)	Tendon disorder	776 (5.72)
Europe	1350 (25.21)	Muscular weakness	541 (3.99)
Asia	22 (0.41)	Musculoskeletal pain	479 (3.53)
Africa	6 (0.11)	Muscle spasms	438 (3.23)
Oceania	3 (0.06)	Back pain	358 (2.64)
**Reporting years**		**FQ suspect**^**‡**^	**n = 5558 (100.00)**
1987–1994	32 (0.60)	Levofloxacin	2781 (50.04)
1995–1999	60 (1.12)	Ciprofloxacin	2135 (38.41)
2000–2004	427 (7.97)	Moxifloxacin	287 (5.16)
2005–2009	755 (14.10)	Ofloxacin	176 (3.17)
2010–2014	1296 (24.20)	Norfloxacin	56 (1.01)
2015–2019	2785 (52.01)		

*FQ* fluoroquinolone; *HCP* healthcare professional; *N* total number of reports; n: number of reports in a given category; UN Continent: Continent of the primary source, according to the United nations.

*More than one reporter could be mentioned per individual case;

^†^More than one ADR could be reported per individual case; Data presented for the first 10 ADRs.

^‡^More than one FQ could be reported per individual case; Data presented for the first 5 FQs.

The most frequently reported indication for women was urinary tract infection (N = 594, 18.32%) followed by sinusitis (N = 457, 14.09%), while for men prostatitis was the most reported indication (N = 218, 12.08%) followed by urinary tract infection (N = 171, 9.47%); a total of 1.55% FQs were indicated for prophylaxis. Of note is the fact that more than one indication could have been reported per ICSR.

A total of 13,563 musculoskeletal ADRs (2.53 ADRs per case) were reported in the 5355 included reports with seriousness criteria as *disabling/incapacitating*, or outcome *recovered/resolved with sequelae* or *fatal*. A total of 4750 (88.70%) reports were serious, while 106 (1.98%) were not. The most frequently reported ADRs were arthralgia (N = 2216, 16.34%), tendonitis (N = 1498, 11.04%), pain in extremity (N = 1353, 9.98%), tendon pain (N = 1035, 7.63%) and myalgia (N = 973, 7.17) (Table 1).

Among the total number of 5355 reports included in our analysis, 583 reports had tendon ruptures (N = 534) and/or tendon injuries (N = 107), ADRs reported as PTs included in *Injury, poisoning and procedural complications* SOC.

**Figure 3:**
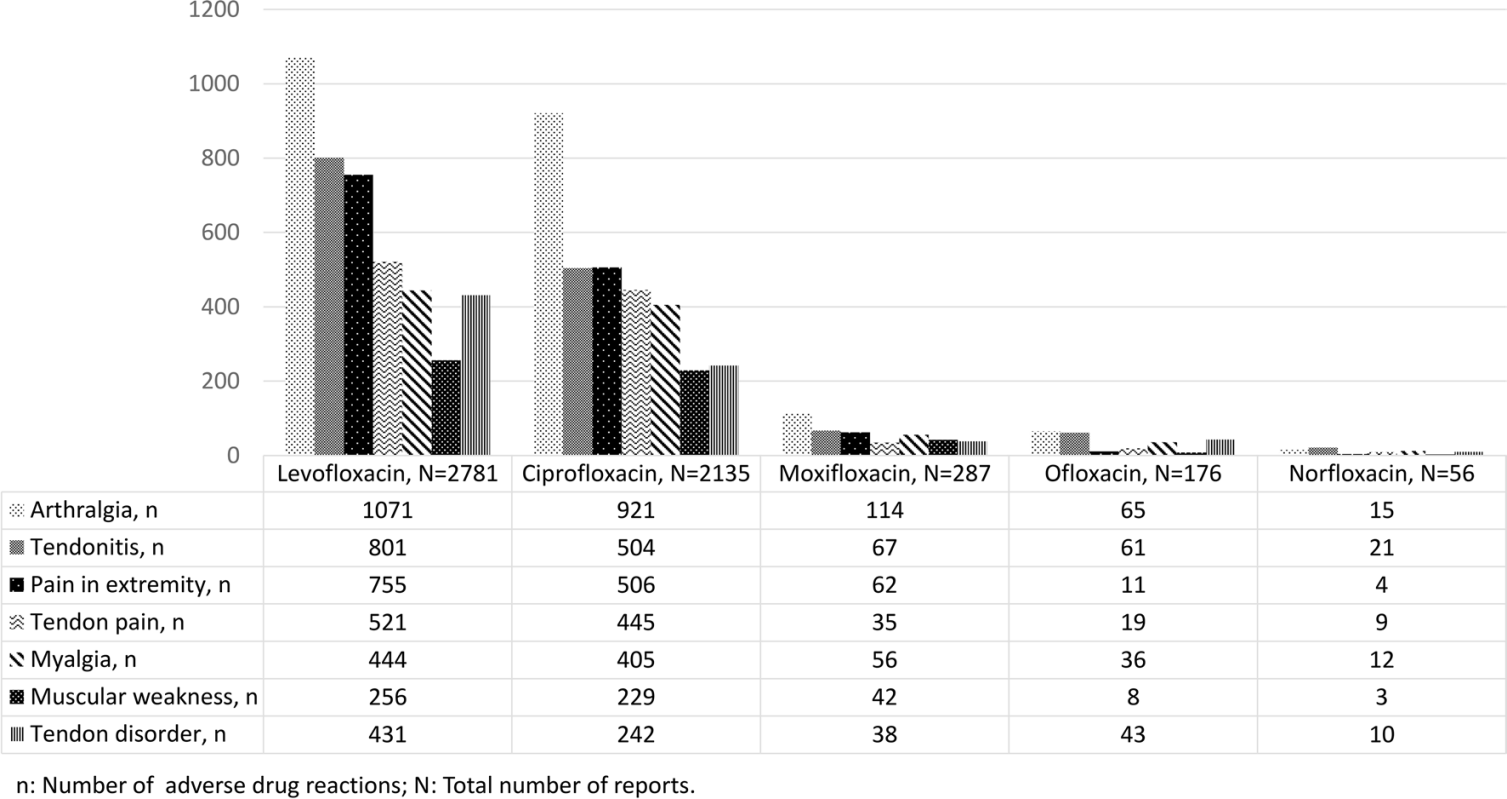
Top most frequent FQs suspected in the reports with musculoskeletal ADRs. *ADRs* adverse drug reactions; *FQ* fluoroquinolone. More than one ADR could be reported per FQ.

The topmost reported FQs were levofloxacin (N = 2781), ciprofloxacin (N = 2135), moxifloxacin (N = 287), ofloxacin (N = 176), and norfloxacin (N = 56) and represented 97% of the total reported FQ-musculoskeletal reports (Table 1, Fig. 3). With respect to the route of administration, the majority of the FQs were administered orally (N = 4202, 57.34%), followed by intravenous administration (N = 193, 2.63%), while a total of 2755 (37.60%) administration routes were unknown.

Dechallenge/re-challenge actions and their outcomes, where available, are presented in Table 2.

**Table 2:** ADRs dechallenge and re-challenge actions and outcomes.

ADRs overall (N = 13,563)			
De-challenge		Re-challenge	
Action N (%)	Outcome n (%)	Action n (%)	Outcome n (%)
Drug withdrawn 3947 (29.10)	No effect observed 2616 (66.28)	Re-challenge 1401 (10.33)	No recurrence 283 (20.20)
	Reaction abated N = 923 (23.38)		Reaction recurred 74 (5.28)
	Fatal 2 (0.05)		Effect unknown 1044 (74.51)
	Effect unknown 406 (10.29)		
Dose not changed 326 (2.40)	No effect observed 115 (35.28)	No Re-challenge 377 (2.78)	Unknown 377 (100)
	Reaction abated 72 (22.09)		
	Fatal 1 (0.31)		
	Effect unknown 138 (42.33)		
Dose reduced 8 (0.06)	No effect observed 3 (37.50)		
	Reaction abated 2 (25.00)		
	Effect unknown 3 (37.50)		

*ADRs* adverse drug reactions; *N* number of ADRs; *n* number of ADRs in a given category.

**Figure 4:**
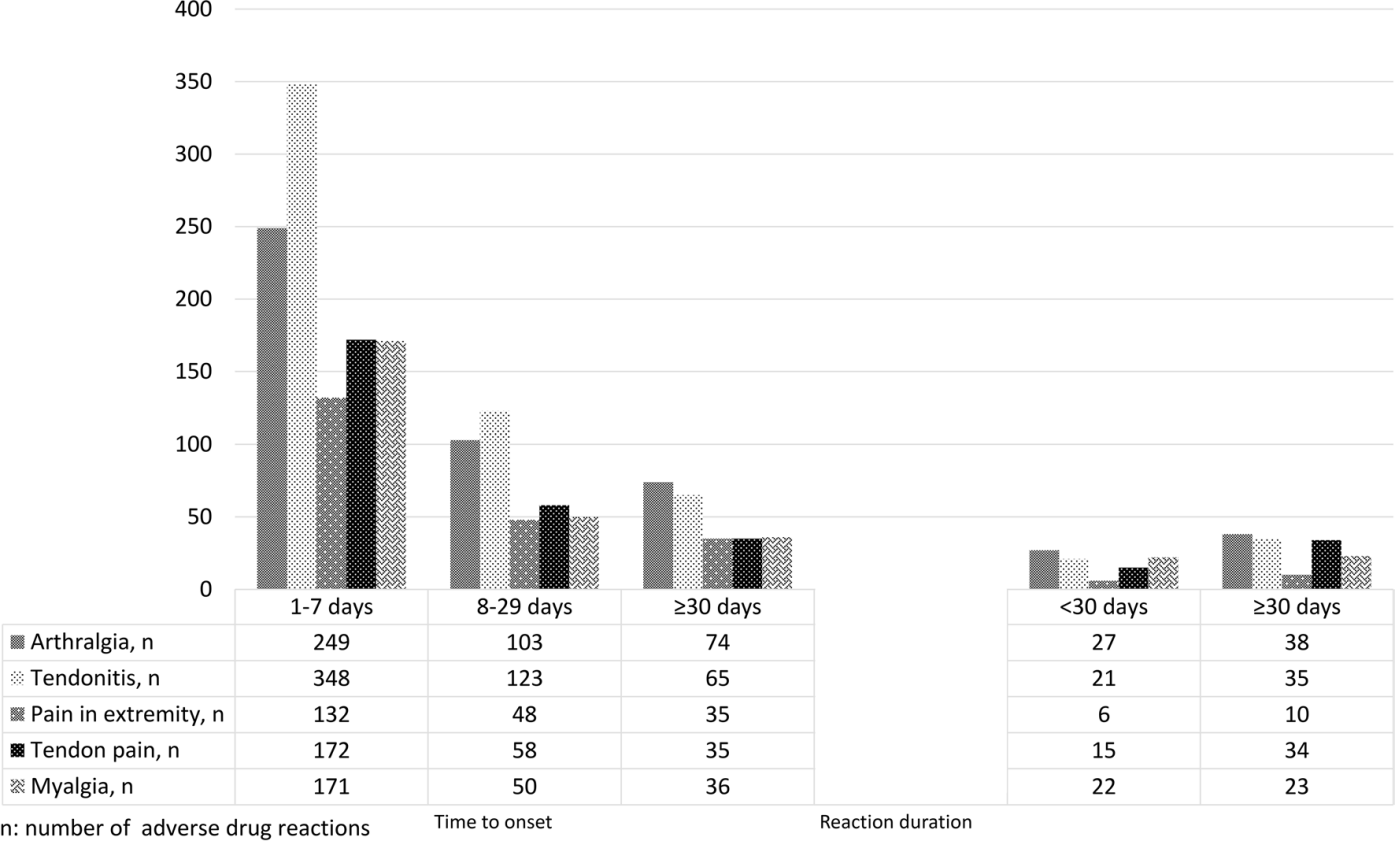
Time to onset and reaction duration of the topmost frequent FQ-musculoskeletal ADRs with available information. *ADRs* adverse drug reactions; *FQ* fluoroquinolone. Data presented for the ADRs that had this information available; More than one ADR could be reported per report.

Overall, the time to onset for the majority of ADRs was between one and seven days (N = 1751, 61.50% from the total N = 2847 ADRs with this data available), and reaction duration exceeded 30 days for most (N = 265, 65.27% from the total N = 406 ADRs with this data available). Figure 4 presents the time to onset and duration of reaction for the top five most reported ADRs, individually.

Among the topmost reported FQs, a total of 2226 unique reports contained 2685 tendon related ADRs, (i.e., tendon discomfort, tendon disorder, tendon pain and tendonitis) linked with N = 2269 FQs administered orally or parenterally.

Among the 2269 FQs, most were administered in a low dose FQ (27.90%), as compared with reports with high doses (5.64%), and this was independent of treatment duration. Overall, the male gender prevailed in ≥ 65 years old age group (N = 311 vs. N = 278), as opposed to female gender being predominant among the 18–64 year-olds (N = 878 vs. N = 552). Corticosteroids were associated with about 7% of FQs, and mostly in the elderly (N = 75 vs. N = 63) (Table 3).

**Table 3:** Tendon related reports with associated risk factors.

	Levofloxacin N = 1208	Ciprofloxacin N = 856	Moxifloxacin N = 82	Ofloxacin N = 91	Norfloxacin N = 32	Overall N = 2269, n (%)
**Dose**						
High dose	107	16	0	5	0	128 (5.64)
Low dose	296	253	35	30	19	633 (27.90)
Unknown	805	587	47	56	13	1508 (66.46)
**Treatment duration ≤ 7 days**						
N:	656	448	43	39	21	1207 (53.20)
Dose: (high/low/unknown)	(67/194/395)	(10/126/312)	(0/23/20)	(3/15/21)	(0/12/9)	(80/370/757)
**Treatment duration > 7 days**						
N:	381	248	23	35	9	696 (30.67)
Dose: (high/low/unknown)	(30/70/281)	(7/78/163)	(0/5/18)	(2/13/20)	(0/6/3)	(39/172/485)
**Treatment duration unknown**						
	171	160	16	17	2	366 (16.13)
**Age ≥ 65**						
N:	405	145	5	32	10	597 (26.31)
Gender: (male/female/unknown)	(212/189/4)	(69/72/4)	(4/1/0)	(20/12/0)	(6/4/0)	(311/278/8)
**Age 18–64**						
N:	668	639	67	46	21	1441 (63.50)
Gender: (male/female/unknown)	(225/439/4)	(269/363/7)	25/42/0)	(27/19/0)	(6/15/0)	(552/878/11)
**Corticosteroids treatment**						
N:	88	49	2	12	1	152 (6.70)
Age (y.o)						
≥ 65/18–64	43/34	25/21	0/2	6/6	1/0	75/63

*N* number of FQs numbered once per report; *n* number of FQs numbered once per report, in a given category; *NA* not applicable; *y.o* years old.

For the top reported FQs, we also looked for disproportionality for the most reported ADRs and for tendon related ADRs in patients who received FQs compared to the reporting of the same ADRs in the full database. Although a positive association for all evaluated FQ-ADR was observed (Table 4), tendonitis, tendon disorder, tendon pain, and tendon discomfort had the highest IC and PRR values for all top FQs.

**Table 4:** Disproportionality data analysis.

	N	IC (95% CI)	PRR (95% CI)
**Levofloxacin**			
Arthralgia	4231	1.75 (**1.71**–1.80)	3.41 (**3.31**–3.51)
Pain in extremity	2865	1.52 (**1.47**–1.58)	2.90 (**2.79**–3.00)
Tendonitis	4742	6.09 (**6.06**–6.13)	96.65 (**93.50**–99.80)
Tendon pain	1847	6.29 (**6.22**–6.36)	120.88 (**114.32**–127.43)
Myalgia	2373	1.23 (**1.17**–1.28)	2.35 (**2.26**–2.44)
Tendon disconfort	137	5.74 (**5.49**–5.99)	91.45 (**75.07**–107.83)
Tendon disorder	1995	6.27 (**6.20**–6.34)	117.35 (**111.25**–123.45)130
**Ciprofloxacin**			
Arthralgia	3504	1.26 (**1.22**–1.31)	2.42 (**2.34**–2.50)
Tendonitis	2714	5.07 (**5.01**–5.12)	40.38 (**38.77**–41.99)
Pain in extremity	2054	0.82 (**0.76**–0.89)	1.78 (**1.70**–1.85)
Myalgia	2021	0.78 (**0.71**–0.84)	1.72 (**1.65**–1.79)
Muscular weakness	880	0.97 (**0.87**–1.06)	1.96 (**1.84**–2.09)
Tendon discomfort	130	5.48 (**5.23**–5.73)	73.09 (**59.80**–86.38)
Tendon disorder	920	4.94 (**4.84**–5.04)	36.59 (**34.12**–39.06)
Tendon pain	1475	5.75 (**5.68**–5.82)	75.11 (**70.77**–79.45)
**Moxifloxacin**			
Arthralgia	865	0.70 (**0.61**–0.80)	1.63 (**1.53**–1.74)
Tendonitis	514	4.11 (**3.99**–4.24)	18.14 (**16.62**–19.66)
Tendon disorder	195	4.12 (**3.91**–4.33)	18.71 (**16.23**–21.19)
Myalgia	569	0.41 (**0.29**–0.53)	1.33 (**1.22**–1.43)
Tendon pain	285	4.8 (**4.63**–4.97)	30.75 (**27.31**–34.20)
Tendon discomfort	13	3.29 (**2.40**–4.18)	15.19 (**8.75**–21.63)
**Ofloxacin**			
Arthralgia	521	0.60 (**0.48**–0.73)	1.52 (1.40–1.64)
Tendonitis	674	5.12 (**5.01**–5.23)	37.23 (**34.51**–39.96)
Tendon disorder	260	5.13 (**4.95**–5.31)	39.09 (**34.54**–43.63)
Myalgia	422	0.61 (**0.46**–0.75)	1.52 (**1.39**–1.66)
Tendon pain	113	4.06 (**3.78**–4.34)	18.28 (**15.18**–21.38)
Tendon discomfort	12	3.55 (**2.62**–4.48)	21.67 (**12.22**–31.12)
**Norfloxacin**			
Tendonitis	369	5.04 (**4.89**–5.19)	35.18 (**31.78**–38.59)
Arthralgia	235	0.27 (**0.08**–0.46)	1.21 (**1.06**–1.35)
Myalgia	209	0.41 (**0.20**–0.61)	1.33 (**1.16**–1.49)
Tendon disorder	54	3.61 (**3.20**–4.03)	13.79 (**10.55**–17.02)
Tendon pain	55	3.76 (**3.36**–4.17)	15.49 (**11.88**–19.09)
Tendon discomfort	11	3.81 (**2.83**–4.79)	34.89 (**19.20**–50.58)

Bolded text represents IC025 and PRR025, the lower ends of the 95% respective confidence intervals.

*CI* confidence interval; *IC* information component; *N* number of reports; *PRR* proportional reporting ratio.

## Discussion

The present study is, to the best of our knowledge, the first to evaluate the case reports with FQ-associated disabling musculoskeletal ADRs, as reported in VigiBase, the unique WHO global database of ICSRs. The study augments the review performed by EMA and FDA. Generally, VigiBase includes more data when compared to those of one or more countries.

The majority of the cases included in our analysis were reported in America and Europe, while a notably lower number was reported in other UN continents. An increase in the frequency of reporting has been observed, with a peak reached in 2017, followed by a slow decrease until 2019. This could be associated with a Weber-like effect triggered by the regulatory activities raising awareness on safety issues, such as the safety communication made by the FDA in December 2016 and the start of the referral procedure in EU early 2017, thus resulting in a peak in reporting followed by a continuous decline thereafter^20^.

Similar to a summary of reports in the FDA’s adverse event reporting system which found that 3% of the FQ-induced tendon-rupture reports were for FQs used for prophylaxis^21^, we too observed that 1.55% of the FQs reported in VigiBase were indicated in prophylaxis. This aspect could in fact hypothesise a pattern of unnecessary overuse in the context of uncertain indication of use or prophylaxis.

Consumers were the most frequent reporters, followed by physicians and pharmacists. A recent analysis of voluntary reports of ADRs in the United States (US) noted an increase in those notified by consumers since 2013^22^. Our analysis showed an increase in consumers/ non-HCPs reports, and overall, these represented the majority of the cases, reflecting the impact these ADRs have on the consumer's quality of life (QoL)^23–25^, in addition to musculoskeletal ADRs being more easily identifiable^23^. Patient reports usually provide more information on the severity of the experienced events as well as the impact on their QoL^24^ and tend to report disabilities more often than HCPs^23^. Based on this, our selection criteria (i.e., disabling/incapacitating ADRs) would in fact favor patient-reported cases.

Several case reports have revealed an association between FQ use and tendinopathies (tendinitis, tendinosis)^12,26^, with more than 40 cases reported already between 1992 and 1994^27^. As a result of their expanded use, an increase in the reports of FQ-associated tendinitis with and without rupture has been observed^26^.

Arthralgia, tendonitis, pain in extremity, and tendon pain were among the topmost frequently reported ADRs in our analysis, in accordance with the latest literature claims^26,28–30^. FQ-induced tendon damage is thought to be linked to oxidative stress and mitochondrial toxicity^31^, and although the exact pathophysiology of FQ-induced tendon injury is not entirely understood, the negative effect of FQs on tendons is likely multifactorial^32^. Due to their chelating proprieties, FQs can interact with regulating proteins of tendinocites, thus damaging the tendon structure. In addition to this, the final event in the pathogenetic mechanism has been suggested to be apoptosis^26^. Direct toxicity and degenerative changes in collagen fibers as a potential mechanism for FQ-induced tendon disorders have also been described^29^.

FQs most frequently linked with ADRs were ciprofloxacin, levofloxacin, moxifloxacin, and ofloxacin. Most of the reports originated from the US; therefore, prescription and utilization patterns of specific quinolone antibacterials might impact the type of FQ most frequently reported. A study on trends in utilization in the US found that more than 80% of quinolone antibacterials prescriptions reimbursed by Medicaid were for ciprofloxacin and levofloxacin, followed by ofloxacin^33^. Also, for example, in the UK, ciprofloxacin, levofloxacin, ofloxacin, and moxifloxacin are the only FQs licensed for use^34^.

Animal models revealed that each quinolone possesses a different potential to cause cartilage toxicity; however, when given a sufficiently high exposure, cartilage changes will occur with all quinolones^35^. For tendon rupture risk, a study found that it was not apparently different among the FQs^36^. Among the top FQs in our analysis, no significant differences were observed. Of note is the fact that our data is based on a spontaneous reporting system (SRS), therefore, a direct comparison among the FQs was not possible nor intended.

In terms of age, even though pain incidence^37^ and myopathies are more likely to occur in the elderly population^38^, we found that patients between 18 and 64 years old were subject of over half of the case reports, while patients aged 65 or older, were involved in just about a quarter. The relatively important percentage of younger subjects might be explained by the fact the elders might be less likely to report ADRs^23^. The use of FQs in elderly and younger populations has been evaluated in comparative studies, and even though age per se did not seem to negatively impact the safety of these antibacterials, special consideration must be given to specific ADRs when bacterial infections therapy is chosen^39^. Renal function declines consistently with age and consequently, doses of renally excreted quinolones (e.g., ofloxacin, levofloxacin) need to be adjusted in case a clinically relevant reduction of creatinine clearance is identified^39^. Moreover, age older than 60, aside from male gender, renal dysfunction, or transplant, were described in literature among the predisposing factors involved in FQ-induced tendinopathy^40^. In terms of gender, similar to a UK study reporting that the detrimental effects of quinolones, such as tendon disorders, are more pronounced among women^41^, in our analysis, the female gender prevailed as well. This might be a real association, or might be linked to a higher likelihood of reporting of ADRs by the female gender^23^.

FQ-induced tendon disorders were reported to have occurred soon after the exposure, with tendon ruptures within one week of administration^10^ and tendinopathies within the first month^32^. Similarly, we too, observed that the time to onset for the topmost frequent FQ-musculoskeletal ADRs was less than 7 days, with the majority in the first month, while the duration of reaction exceeded 30 days for most cases. However, it should be mentioned that a very limited number of reports had this information available. Also, our initial selection of disabling/incapacitating or recovered/resolved with sequelae might explain the slightly higher number of long-lasting or prolonged (≥ 30 days) ADRs.

With regards to de/re-challenge actions, a low number of reports had this information available. In almost a quarter of the ADRs with drug subsequently withdrawn, the reaction abated, and in more than half, no effect was observed. Similarly, from the ADRs with dose kept the same, generally, no effect was observed. No recurrence of the reaction was observed in the majority of the ADRs where re-challenge was performed. However, the scarce data available regarding de/re-challenge actions (60%/ 80% of the total number of ADRs did not have this information available) may weaken the possibility of generating any hypothesis based on this data. Moreover, through the prism of the selection criteria, cases with a poor outcome have been favored for inclusion in the analysis, while positive outcomes might have been missed (i.e., reaction abated), thus explaining the prevalence of the former.

Most of the literature evidence points to a tendon related risk associated with FQs, with different potential associated risk factors. Consequently, we limited the number of reports for which we looked for these risk factors strictly to reports of tendon related disorders (2226 unique reports).

A notable risk of tendon disorders was found to be associated with the use of systemic corticosteroids^36^, especially in combination with current exposure to FQs, when the risk of tendon rupture/injury was found to be strongly increased^11,40^. For the top FQ reports with tendon related ADRs, a low number had concomitant corticosteroid use reported (6.70%). Among these, a slightly increased corticosteroid exposure was observed in patients older than 65 years old, as compared to younger patients (49.34 vs. 41.45).

A systematic review of observational studies noted that advanced age might constitute a risk factor for FQ-induced tendon disorders^32^, and a critical review of literature observed a ratio of men to women of 1.9:1 for tendinopathies secondary to FQs^42^. In our analysis, although only a few cases with both advanced age and male gender were identified, we did notice a predominance for male gender among the ≥ 65 years old age group, possibly suggesting that after the age of 65, men could be more predisposed to tendon disorders. The same systematic review pointed out that higher FQ dosages may be associated with higher risk of tendon disorders^32^. From the reports evaluated for risk factors, we did not find any indication pointing to high doses being a contributing factor. Moreover, treatment duration, where available, was less than 7 days in most reports, suggesting that a prolonged FQ treatment duration might not contribute to risk potentiation.

An added greater risk for tendon disorders could also be linked with obesity in the context of mechanical forces exerted on body structures during activity^41^ as well as diseases like diabetes, renal impairment^32,41^, or organ transplant^41^. Unfortunately, in the context of the dataset we based our analysis on, comorbidities could not be thoroughly depicted nor described.

With regards to disproportionality data analysis performed overall, on the total number of musculoskeletal ADRs, for the top FQs, all evaluated ADRs had PRR025 value ≥ 1, associated with ≥ 5 case reports, and were therefore considered positive associations. Similarly, the IC025 value (the lower end of the CI for IC) was ≥ 0 in all cases, indicating a positive FQ-ADR association. Tendonitis, tendon disorder, and tendon pain had the highest IC and PRR values.

### Limitations

A variation between reports and submitting countries exists with regards to the amount of information reported. Causality can also vary from report to report. Some countries collect only suspected ADRs with at least a possibility of a causal relationship between the drug and reported event, whilst for example, the US (contributing almost half of the reports in VigiBase) collects "any adverse event associated with the use of a drug in humans, whether or not considered drug related"^14^. As with all SRSs, limitations are mainly related to under-reporting and variable quantity and quality of the reported data^43^. Another notable limitation of SRS, for our study was the low availability of data regarding concomitant medication. Therefore, this did not allow to evaluate the contributory role of corticosteroids use described in literature as potential potentiator of tendon disorder risk among patients with concomitant FQ and corticosteroid use.

Through the prism of selecting reports pertaining exclusively to *musculoskeletal* SOC, we might have missed ADRs in terms of PTs, such as tendon rupture and injury, included in other SOCs (e.g., *Injury, poisoning and procedural complications *etc.). However, we aimed to evaluate ADRs strictly related to FQ induced musculoskeletal system disorders, and avoid possible confounding factors like for example situations where injuries, procedural, or device complication factors could be significant in the medical event being reported (as is the case for *Injury, poisoning and procedural complications* SOC).

The present analysis might also be limited by the selection of disabling/incapacitating or recovered/resolved with sequelae or fatal ADR reports, which could have impacted the results in terms of proportion of long-lasting ADRs, de/rechallenge outcomes, and may have even added bias with regards to predominant notifier type. However, since the safety concerns targeted long-lasting, disabling, and potentially irreversible ADRs, we considered these to be of primary interest.

Moreover, we only selected cases where an FQ was the suspect drug, thus excluding reports with interacting FQs. By doing so, we aimed to avoid reports where ADRs might have been induced by other drugs or that might have had other alternative causes. The objective was to analyze cases where a clear FQ-ADR association was assumed, with as little confounding factors as possible.

## Conclusions

The present analysis of musculoskeletal disorders associated with FQs in VigiBase revealed that the majority of the cases were reported by consumers/non-HCPs, with an indicated ascending trend of reporting. The female gender and 18–64 years old age group prevailed. The topmost reported ADRs (arthralgia, tendonitis, pain in extremity, tendon pain, myalgia) and FQs (levofloxacin, ciprofloxacin, moxifloxacin, ofloxacin, norfloxacin) corroborate the musculoskeletal safety concerns recently evaluated by the authorities, also supported by the disproportionality data.

## Data Availability

The data that support the findings of this study are available from UMC but restrictions apply to the availability of these data, which were used under license for the current study, and so are not publicly available. Data are however available from the authors upon reasonable request and with permission of UMC.
